# Defining petal cell identity layer-by-layer

**DOI:** 10.1093/plcell/koad283

**Published:** 2023-11-07

**Authors:** Humberto Herrera-Ubaldo

**Affiliations:** Assistant Features Editor, The Plant Cell, American Society of Plant Biologists; Department of Plant Sciences, University of Cambridge, Cambridge CB2 3EA, UK

Flowers' great diversity and beauty are explained by extensive variation in petal color, shape, and pattern. The combination of colors and sizes in the flower organs is key to attraction and interaction with pollinators, hence affecting reproduction. At the molecular level, the formation of floral organs is guided by the combinatorial action of transcription factors as illustrated by the classical ABC model (Coen and Meyerowitz 1991). Petal identity is controlled by the B-class proteins, for example, APETALA3 and PISTILLATA in *Arabidopsis* or DEFICIENS and GLOBOSA in *Antirrhinum* (Purugganan et al. 1995); then other transcription factors acting downstream control pigmentation, color patterns, and other special features in the petals (Kellenberger et al. 2023). Our understanding of the molecular mechanisms guiding floral diversity is limited.

In this issue of *The Plant Cell*, **Mathilde Chopy and colleagues (Chopy et al. 2023)** dissected the function of the B-class protein DEFICIENS during petal development in petunia flowers and identified cell layer–specific mechanisms that guide the formation of the corolla tube and limb. In Petunia, there are 4 B-class proteins, PhDEFICIENS and PhTM6 (DEF/AP3 clade) and PhGLO1 and PhGLO2 (GLO/PI clade). Double mutants in B-class genes have the classical B-function mutant phenotype: petals are converted into sepals and stamens into carpels. In contrast, the functions of the DEF/AP3 clade have diverged. PhDEF works in the petal, whereas PhTM6 (in combination with C-class proteins) functions in stamen development. The single mutant *phdef* displays homeotic conversion of the petal whorl into sepals; in contrast, stamen development is unaltered (Rijpkema et al. 2006).

The petunia flower has 5 fused petals with 2 well-defined domains: a flat, wide, and colorful limb and a long tubular structure called the tube (see Fig.); variations in tube length can affect its interaction with pollinators. The *phdef-151* allele is a null mutation caused by the insertion of the dTph1 transposon in the *PhDEF* locus. In the *phdef-151* mutant, the petals are converted into sepals; however, in some cases, the authors observed recovery as well as a restoration of petal development in some branches of the *phdef-151* plants. They classified the recovery events into 2 classes, *wico* and *star*, with contrasting features. The *wico* mutant displayed nearly normal corolla development, but the tube was underdeveloped, showing a reduction of about 3-fold in length. On the other hand, the *star* mutant showed strongly reduced lateral expansion, causing a 5-fold decrease in the limb area; the tube displayed a slight reduction in length. In summary, the tube and limb development programs could be uncoupled.

**Figure.**
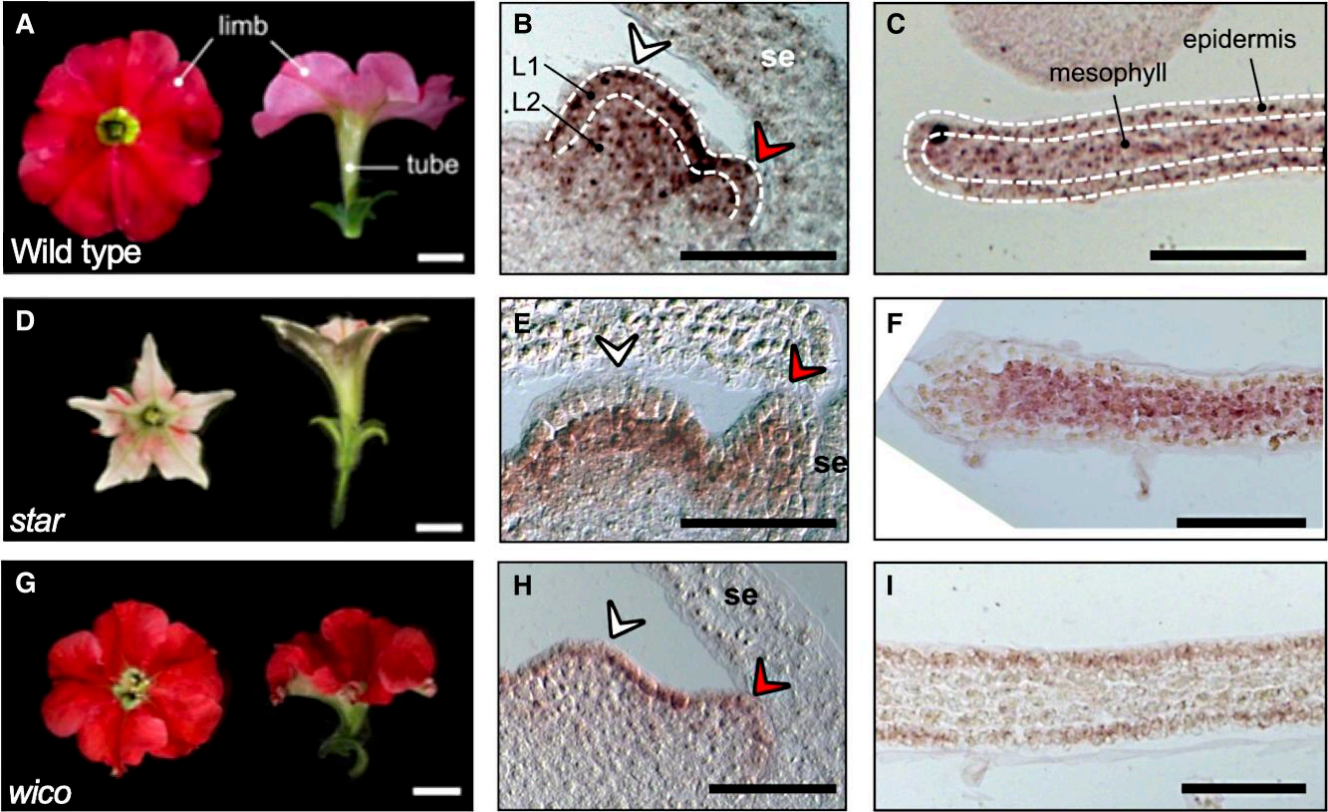
Layer-specific expression of *PhDEFICIENS* guide limb and tube development in petunia flowers. Top and side view of wild-type **(A)**, *star***(D)**, and *wico***(G)** flowers. Bars = 1 cm. Localization of the *PhDEF* transcript in wt, *star*, and *wico* flower meristem cell layers **(B**, **E**, **H)** and the young petal limb **(C**, **F**, **I)**. White and red arrows indicate stamen and petal primordium, respectively; se, sepal; L1, layer 1; L2, layer 2. Scale bar = 50 *μ*m. Adapted from Chopy et al. (2023), Figures 1 and 3.

The recovery of petal formation in some presumed *phdef-151* mutants suggested that phDEF activity was reactivated, probably mediated by transposon movement out of the *PhDEF* locus. The rearrangement events were analyzed by sequencing the *PhDEF* locus in the *wico* and *star* mutants; surprisingly, they found similar sequences, either wild type or sequences with +6 base pairs producing 2 extra amino acids in PhDEF. Because there was no difference at the sequence level, the authors analyzed the *PhDEF* transcript distribution using in situ mRNA hybridization. The *PhDEF* transcript has a wide distribution across the different cell layers in the wild-type flower meristems (L1–L3). However, in the *wico* mutant, the signal was detected only in the L1; conversely, in the *star* mutant, the transcript was detected in the L2 and L3. These results indicate the function of PhDEF can be spatially modular: its transcript presence in the L1 drives the development of the limb, whereas its presence in the L2 drives the development of the tube. In summary, the expression of *PhDEF* in the petal epidermis is the main driver of limb morphogenesis and controls features such as growth, shape, and pigmentation, whereas mesophyll drives tube morphogenesis.

Next, analyses were conducted to study cell identity across the petal cell layers. The wild-type adaxial limb cells are conical, whereas the tube cells are elongated. In *wico*, the limb cells remain conical but bigger, whereas cells in the tube are similar in shape but shorter. Cells in the *star* limb are rounder, and cells in the tube are similar but shorter. Interestingly, in *star* the epidermal cells in the limb have an intermediate identity between petal and sepals. The layer-specific expression of *PhDEF* in *wico* or *star* lines is a good system to study the function of *PhDEF* in the cells where it is expressed (cell-autonomous function) and the role in the neighbor layers (non-cell autonomous).

Finally, to look at the processes downstream of *PhDEF*, the authors conducted gene expression analysis through petal development in the wild-type, *phdef-151*, *wico*, and *star* lines. They focused on differentially expressed genes related to the production of pigments. Comparison between the *phdef-151* or *star* and the *wico* expressed genes indicated a strong downregulation of genes in the anthocyanin biosynthesis pathway. Interestingly, they identified some genes encoding major regulators of petal pigmentation, in particular, some members of the MBW (MYB, bHLH, WD40) complex. They found the predicted PhDEF DNA-binding sequence in the *ANTHOCYANIN1* (*AN1*) and *AN2* regulatory regions. The binding events were tested using electro-mobility shift assays; in vivo analysis found PhDEF directly binds to the terminator region of *AN2*, indicating PhDEF could directly control pigmentation in the petal limb epidermis.

In summary, the B-class protein PhDEFICIENS has layer-specific functions during petal development. In the epidermis, PhDEF has a role in the control of petal pigmentation, conical cell shape, and limb growth, whereas the expression in the mesophyll directs tube growth.

## Dive Curated Terms

The following phenotypic, genotypic, and functional terms are of significance to the work described in this paper:

petal AmiGo: PO:0009032
